# Supplementary material for: A general model unifying the adaptive, transient and sustained properties of ON and OFF auditory neural responses
Source: PLoS Comput Biol. 2024 Aug 2;20(8):e1012288. doi: 10.1371/journal.pcbi.1012288 (PMC11324186; doi:10.1371/journal.pcbi.1012288)
Supplement: S1 Note — We show here how to mathematically derive analytical formulas for the transfer functions, magnitude-frequency curves, poles and zeroes, as well as a recursive formulation of our AdapTrans filters. (PDF) [file pcbi.1012288.s001.pdf]

Supplementary Note 1: Mathematical derivation of AdapTrans properties

Transfer functions

We demonstrate here how to compute the formulas for the transfer functions of Eq.4. Since the principle is the same for each polarity, we only provide the example of the ON channel. The transfer function is the Z-transform of the discrete-time impulse response (Eq.1):

$$
\begin{aligned}
H_{ON}(z) = Z\{h_{ON}[n]\} &= \sum_{n=0}^{+\infty} h_{ON}[n]z^{-n} \\
&= h[0] + \sum_{n=1}^{+\infty} h_{ON}[n]z^{-n} \\
&= 1 - Cw\sum_{n=1}^{+\infty} a^{n-1}z^{-n} \\
&= 1 - Cwa^{-1}\sum_{n=1}^{+\infty} (\frac{a}{z})^n \\
&= 1 - (Cwa^{-1}\sum_{n=0}^{+\infty} (\frac{a}{z})^n - Cwa^{-1}) \\
&= 1 + Cwa^{-1} - Cwa^{-1} \times \frac{1}{1-\frac{a}{z}} \\
&= \ldots \\
&= \frac{1-(a+w-aw)z^{-1}}{1-az^{-1}}
\end{aligned}
\tag{S1}
$$

Magnitude-frequency curves (Bode)

From the expression of the transfer function, the formula for the magnitude-frequency response (expressed in dB) follows:

$$
\begin{aligned}
A_{ON}^{dB}(\omega) = 20\log(|H_{ON}(e^{j\omega})|) &= 10\log(|H_{ON}(e^{j\omega})|^2) \\
&= 10\log(\frac{|1-(a+w-aw)e^{-j\omega}|^2}{|1-ae^{-j\omega}|^2}) \\
&= 10\log(\frac{|(1-a+w-aw)\cos(-j\omega)-\sin(-j\omega)|^2}{|(1-a)\cos(-j\omega)-a\sin(-j\omega)|^2}) \\
&= 10\log(\frac{((1-a+w-aw)\cos(\omega))^2+((a+w-aw)\sin(\omega))^2}{((1-a)\cos(\omega))^2+(a\sin(\omega))^2}) \\
&= \ldots \\
&= 10\log(\frac{1+(a+w-aw)^2-2(a+w-aw)\cos(\omega)}{1+a^2-2a\cos(\omega)})
\end{aligned}
\tag{S2}
$$

Poles and zeroes

AdapTrans filters have one pole and one zero, which can be read directly from the expression of the transfer function. For instance, in the case of the ON polarity, the pole is a and the zero is $a+w-aw$.

Recursive equation

From the expression of AdapTrans transfer functions, we can derive their corresponding discrete-time recursive equation:

$$
\begin{cases}
y_{ON}[n] = x[n]-(a+w-aw)\times x[n-1]+a\times y_{ON}[n-1] \\
y_{OFF}[n] = -w\times x[n]+(1-a+aw)\times x[n-1]+a\times y_{OFF}[n-1]
\end{cases}
\tag{S3}
$$

This recurrent formulation has the advantage of implementing an actual IIR filter, rather than a long FIR filter. IIR filters are particularly suited for real-time online processing on dedicated hardware such as Digital Signal Processors (DSPs) or Field-Programmable Gate Arrays (FPGAs).
