# Supplementary figures and images for: A general model unifying the adaptive, transient and sustained properties of ON and OFF auditory neural responses
Source: PLoS Comput Biol. 2024 Aug 2;20(8):e1012288. doi: 10.1371/journal.pcbi.1012288 (PMC11324186; doi:10.1371/journal.pcbi.1012288)

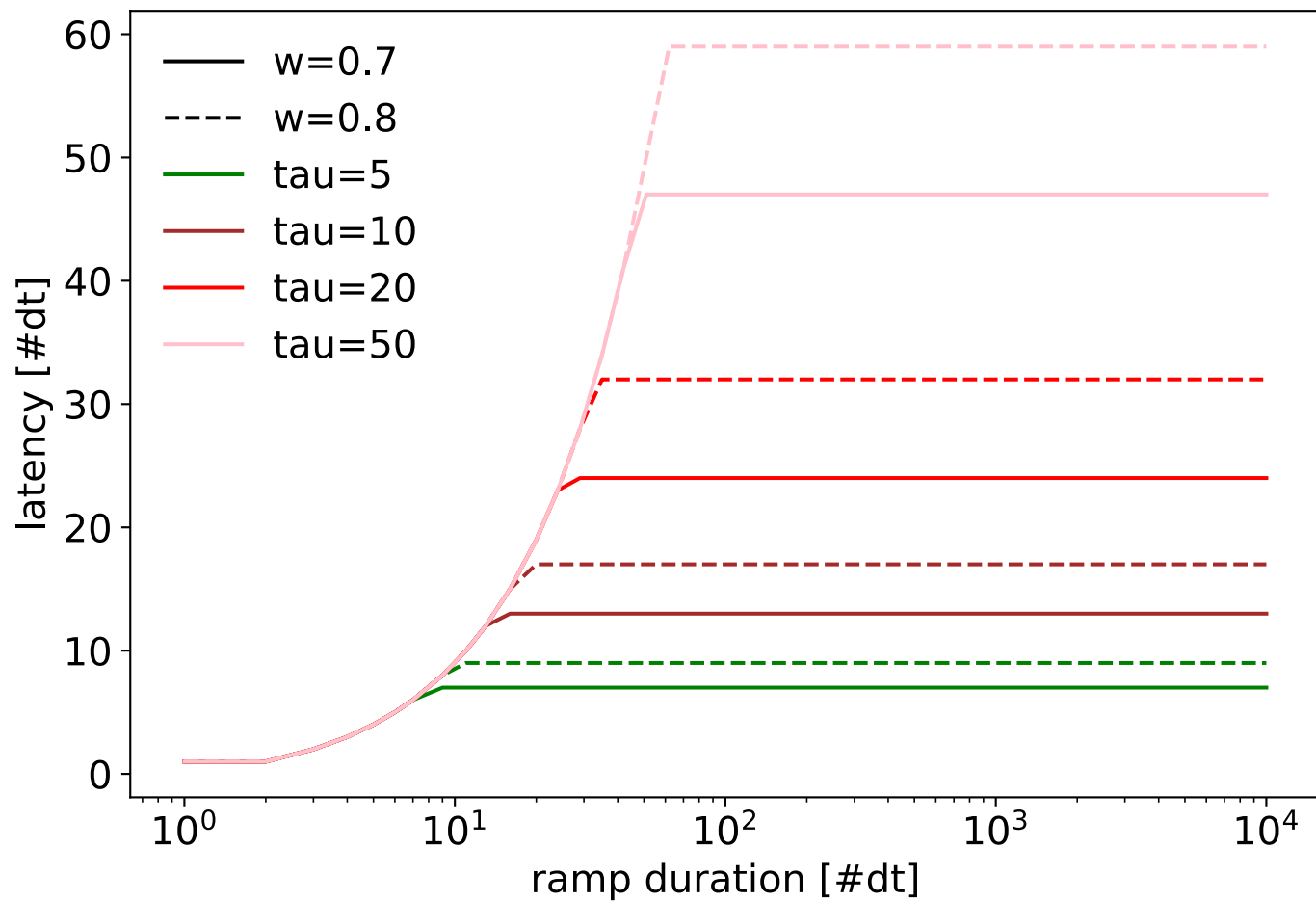

Supplement: S1 Fig — The simulated experimental setup described in Fig 4A allows to extract the AdapTrans offset response latency as a function of the ramp duration. S1 Fig below shows this relationship for different pairs of w parameter and OFF time constant (tau). Our model predicts that latency increases as fall ramps become less abrupt. To our knowledge, this has never been tested by electrophysiologists. We hope that future experimental studies will address this question. (PDF) [file pcbi.1012288.s003.pdf]

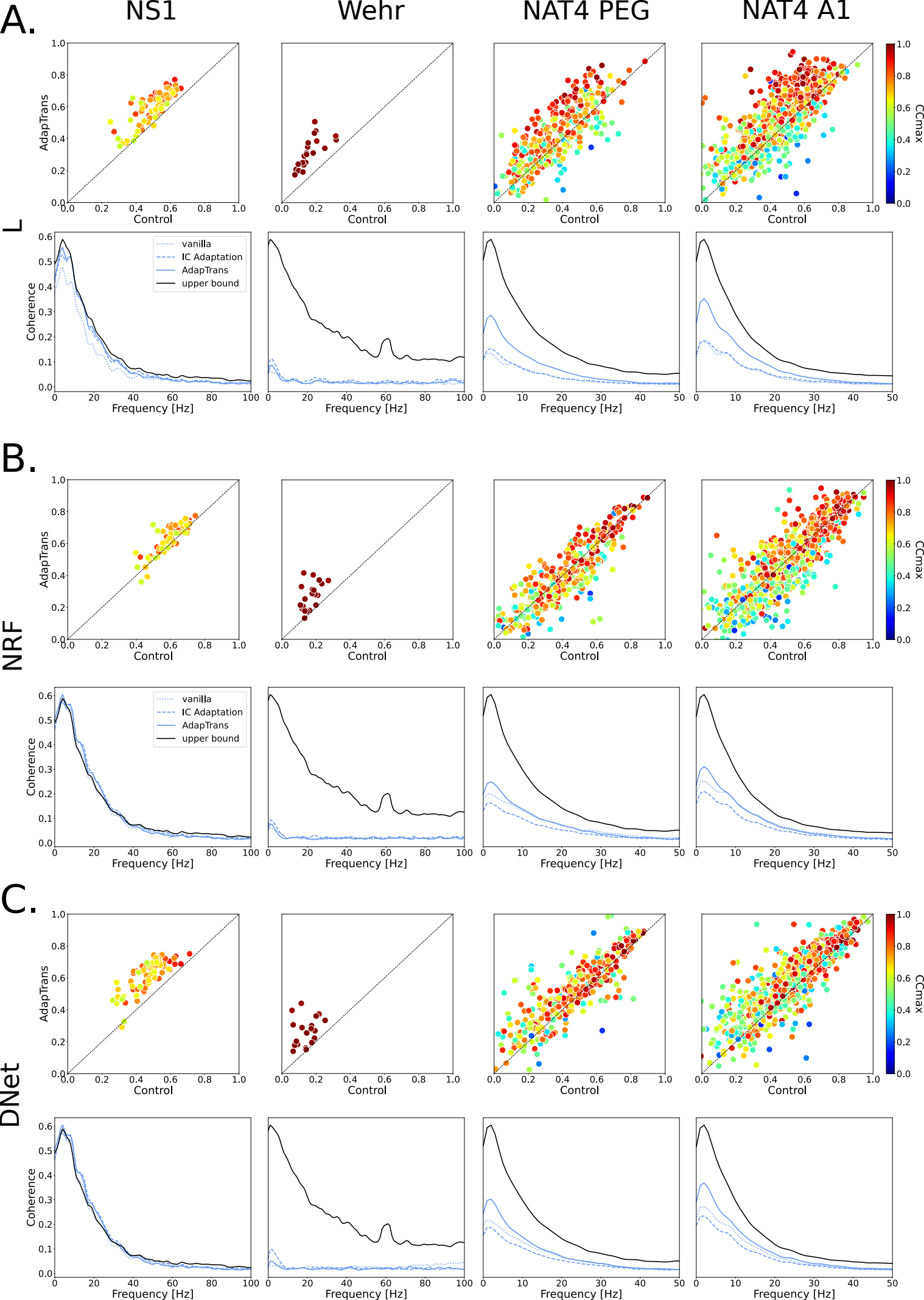

Supplement: S2 Fig — We present below the scatter and coherence plots for the L, NRF and DNet models on all datasets. (PDF) [file pcbi.1012288.s004.pdf]

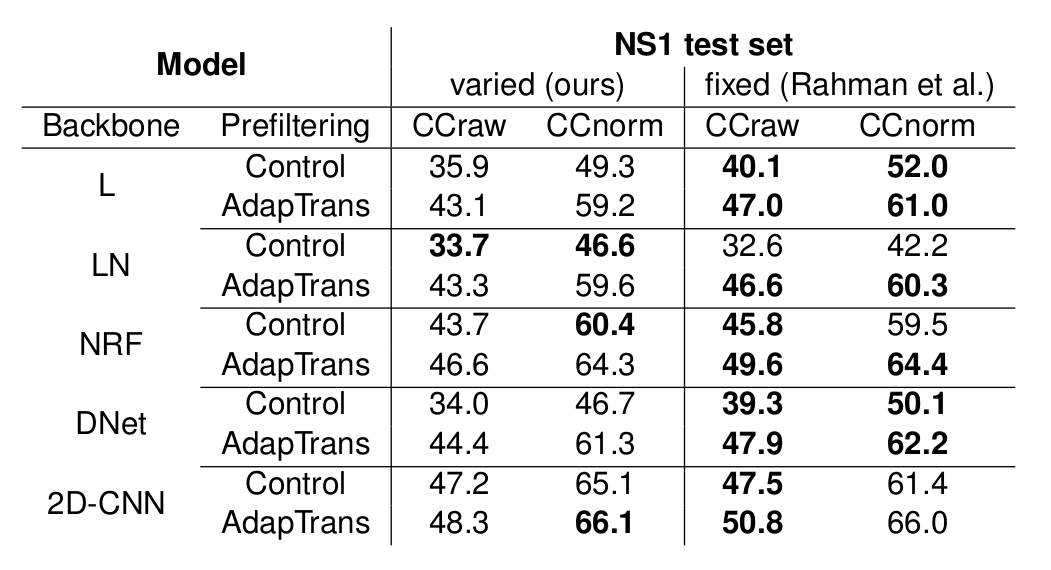

Supplement: S1 Table — In [50], a fixed data subset was held-out during model development, and “opened” back only for testing models, while various splits of the remaining data were used for training and validation. This strategy might be risky if the selected dataset is significantly easier or harder than the training and validation sets, and results in over or under-estimated performances. The table presents Test performances of models depending on the cross-validation methodology. Models and the rest of the training pipeline were identical for both conditions. Performance metrics are the correlation coefficient and normalized correlation coefficient, in %. (PNG) [file pcbi.1012288.s005.png]

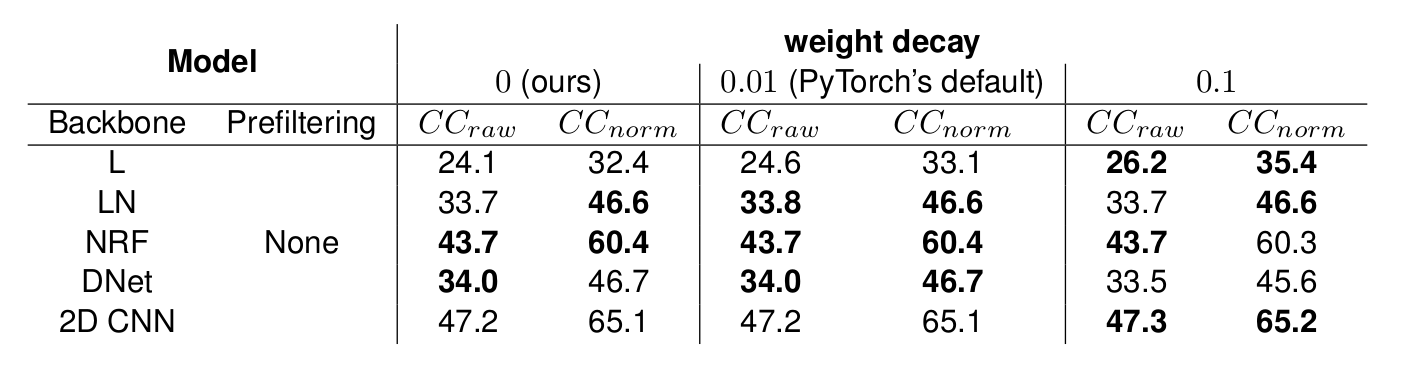

Supplement: S2 Table — The auditory response fitting literature has a long tradition of explicit model regularization in the form of L1 or L2 penalty on STRF weights. However, initial models were fitted via zero-order algorithms such as boosting, and not gradient descent (as in this study), an ubiquitous optimization algorithm which may display some intriguing implicit regularization [51]. Following innovations from the machine learning community, we have incorporated batch normalization in our models, and followed a rigorous fitting procedure with separate training and validation sets and an early stopping criterion. During our initial model development phase, we investigated whether the weight decay readily available in PyTorch could lead to better performances. We report here results of control models on the NS1 dataset. The table presents test performances as a function of the weight decay parameter used with AdamW optimizer in our setup. Models and the rest of the training pipeline was identical for both conditions. Performance metrics are the correlation coefficient and normalized correlation coefficient, in %. (PNG) [file pcbi.1012288.s006.png]
