# Supplementary material for: A general model unifying the adaptive, transient and sustained properties of ON and OFF auditory neural responses
Source: PLoS Comput Biol. 2024 Aug 2;20(8):e1012288. doi: 10.1371/journal.pcbi.1012288 (PMC11324186; doi:10.1371/journal.pcbi.1012288)
Supplement: Attachment — Submitted filename: AdapTrans_PLoS_CB_response_letter.pdf [file pcbi.1012288.s007.pdf]

We thank the two reviewers for their positive evaluation of our paper as well as their very helpful suggestions and insights. We hope that we correctly addressed their comments in this revised version, re-submitted on BioRxiv.

Before they consult our responses to their particular questions/remarks, we would like to notify both reviewers of the following changes:

- The Wehr dataset is especially interesting because of its nature: it is composed of neuronal membrane potentials obtained via patch-clamp (current-clamp mode), not (extracellular) spikes. However, unlike spikes, this kind of signal can be subject to drift, and we found that preprocessing recorded neural responses with a linear detrend greatly helped models to fit the data, especially the simpler ones, like the L model. As a result, all metrics reported from this dataset were obtained with this additional data preprocessing step; the section corresponding to this dataset has also been updated according to the changes above.

- When integrated into larger processing pipelines, our model of auditory ON-OFF responses, AdapTrans, creates a two-channel spectrogram that therefore doubles the number of input weights. As a result, some AdapTrans models can have significantly more parameters than their Control / IC adaptation counterparts (cf. Table 1 in our original submission). Even if the introduction of more parameters due to AdapTrans is not a problem because they remain fully interpretable (i.e., they can be thought of as ON-OFF STRFs), we wanted to compare the prefiltering conditions (i.e., None / IC adaptation / AdapTrans) at iso-parameter counts, whenever possible. As a result, we divided by a factor of 2 the number of hidden units in both AdapTrans NRF and AdapTrans DNet models, effectively reducing the number of free parameters to a similar level as in the Control / IC Adaptation ones. This was unnecessary for the 2D-CNN model, as it is much deeper, and the input layer weighs very little in the total parameter count. Finally, both L and LN models cannot be reduced more, so we did not apply this strategy to the latter. The *“Integration within larger models of Audition”* subsubsection of the *“Materials and Methods”* section has been updated with this information.

- We realized that we forgot to mention our use of Batch Normalization (BN) [Ioffe and Szegedy 2015] in LN, NRF, and DNet models. The latter acts as a source of regularization and joins Reviewer #2 in one of his remarks. We modified the *“Computational Neuron Models”* subsection of the *“Materials and Methods”* section, as well as Figure 2 in this sense.

- A Rectified Linear Unit (ReLU, or “half-wave rectification”) has now been applied to the output of AdapTrans when it was integrated into larger models of audition because we found it to bring a slight improvement in performances on the neural response fitting task. The latter has been added to the “*Materials and Methods”* section, “*AdapTrans model of auditory ON-OFF responses and adaptation”* subsection, “*Integration within larger models of audition”* subsubsection.

All the above changes were also performed on our online code repository.

Reviewer #1

Reviewer #1: This study develops a new front end for central auditory encoding models that explicitly and separately models adapting on and off responses to changes in sound level. The authors incorporated this AdapTrans filter into several published models and tested it on several datasets from A1 and IC. In nearly every case, the addition of AdapTrans improved model performance.

The study was well-motivated by previous work, and the authors were clearly very thoughtful in their approach. The fact that they tested multiple models and datasets is especially novel, and they rightly identify issues around the inability to compare previous studies because of methodological differences. While this work is quite interesting and worth publishing, there are places where the findings are presented in an overly-compressed way, which makes it difficult to fully assess the results. These issues should be addressable.

MAJOR CONCERNS

p. 12. Table 4 provides convincing quantitative evidence for the benefit of the AdapTrans filter, but it is not clear how performance differences are distributed across the set of neurons tested. A scatter plot comparing performance of two models for each neuron clarifies how the improvements were distributed across the populations. Comparing every combination of models might be too much, but a more detailed characterization, for example, of the higher-performing models would help.

Thanks for this suggestion. We now provide in a new figure (now Figure 5) scatter plots showing how improvements in CCnorm obtained with our approach are distributed across the neural population. For each dataset, this is done with the 2D-CNN model (which is the best-performing model across all the data) and also for the LN model because it is frequently used in studies modeling auditory processes. To be exhaustive, we show the scatter plots associated with the other models in the supplements (see supplementary Note

5). These analyses are discussed in a new paragraph of the '*AdapTrans filtering enhances the neural fitting performances of a large spectrum of models*' section of the Results.

p. 14. Somewhat related, the characterization of the key new parameters in Fig. 6 is collapsed across models and datasets. Since data are collected from different species and different behavioral states (awake vs. anesthetized) it is especially important to not collapse across these datasets. Either result would be interesting, whether a and w are consistent or different between datasets.

This is a good point. Figure 7 now provides the distribution of the AdapTrans parameters (i.e., w and the 'ON' and 'OFF' time constants) for each dataset (N.B.: for the NAT4 dataset, we provide the distribution for areas PEG and A1 separately).

p. 13. While it is clear that the new filter improves performance, it is difficult to tell from the example data in Fig. 5 where/how they improve predictions. It does look like peaks in the predicted PSTHs are narrower for the AdapTrans model, which would be consistent with a model that captures transient dynamics more accurately. Can this be quantified? For example, the authors might consider measuring the coherence between predicted and actual PSTHs to see if the benefits are specific to temporal frequency bands. This would help show whether AdapTrans is capturing specific dynamics or just enriching the input space more generally.

This is a very good suggestion. In this new version, we computed the coherence values based on the method described by *Machens et al.,* (2004)(see the new '*Coherence*' subsection in the '*Task and Evaluation of Performance*' section of the Materials and Methods). We showed that AdapTrans also improves this metric, notably in the low frequencies (<10 Hz), suggesting that transitory peaks of activity at these frequencies are better captured. This can be observed in the new version of Figure 6 ('*Various deep learning models fitting auditory neural responses: model prediction versus actual neural activity*') and in a new figure that shows the average coherence values (across the neural population) for each dataset (see new Figure 5). We discuss the results of these coherence analyses in a new paragraph of the 'Results' section (see the '*Coherence analysis*' subsection). In addition, we have shown that capturing OFF responses as early as the cochleagram is the key to success for AdapTrans models, especially on NAT4 datasets, where the so-called "IC Adaptation" typically fails (see Table 3, Figures 5 & 6 and the *"AdapTrans prefiltering enhances the neural response fitting performances of a large spectrum of models"* subsection).

New reference added:

- Machens, C. K., Wehr, M. S., & Zador, A. M. (2004). Linearity of cortical receptive fields measured with natural sounds. *Journal of Neuroscience*, *24*(5), 1089-1100.

LESSER CONCERNS

Minor: It would be helpful if the manuscript could include line numbers.

Sure. Line numbers were added in the new version.

p. 3. "see the 'Model' section". There does not appear to be a section explicitly called "Model". "Computational Neuron Models"?

Ok. This was corrected in the new version.

p. 4. (also p. 15 Discussion) The w parameter provides a creative way to include both sustained and transient information in the stimulus representation. An alternative would be to simply include a third filter bank that does not adapt. Is there a specific theoretical benefit to fusing them in a single filterbank? Numerical efficiency?

This is a very interesting idea. In order to explore whether such an approach leads to better performances, we implemented a new version of our model where we concatenated the raw spectrogram to the adapted ON and OFF channels. Please note that theoretically, AdapTrans parameters can learn *not* to adapt (in this case, the ON kernel become a simple delta function with w=0), but there is no firm guarantee that this can occur in practice, and even if it occurs, it might be better to have both the raw and the adapted signal. As can be observed in the table below, combining both adapted and non-adapted versions of the spectrogram led to better average CCraw and CCnorm in all the datasets on the best-performing model, the 2D CNN inspired by Pennington et al. (2023). Of course, this gain comes at a minor additional computational cost, as suggested by the reviewer.

**Model**		**Dataset**							
		NS1		Wehr		NAT4			
						PEG		A1	
Backbone	Prefiltering	CC_{raw}	CC_{norm}	CC_{raw}	CC_{norm}	CC_{raw}	CC_{norm}	CC_{raw}	CC_{norm}
2D-CNN	AdapTrans	48.3	66.7	26.9	27.4	37.9	52.9	44.3	60.9
	Adap+raw	**48.6**	**67.1**	**28.0**	**28.5**	**38.8**	**54.2**	**45.6**	**62.8**

This improvement of our model is now discussed in a new section of the results ('*Further performance improvements with an augmented AdapTrans scheme and neural population training*'). We provide the table above as new table 4.

p. 5. Table 1. It is not immediately clear what the "F" "T" and "H" headings refer to.

Sorry about that. The hyperparameter *F* refers to the number of frequency bins spanned by each convolutional layer, *T* provides the number of time bins, and *H* the number of hidden units before readout. We have updated the table's caption accordingly.

p. 6. CNN model. An argument of the Pennington and David study was that performance of high parameter count models benefits substantially from fitting to activity of the entire dataset in a single model. Could this architecture have been implemented in the current study?

This is a very interesting question. It is indeed possible to implement such an approach with our model. Nevertheless, it requires the responses of different neural units to the same stimuli, which are not available in the case of the Wehr dataset. Thus we can not use this option "by default". However, to explore this option, we ran new analyses and showed that fitting from the entire neural population significantly improves performances in NS1 and both NAT4 datasets, as can be observed in the table below (Table 5 in the updated manuscript).

**Model**			**Dataset**					
			NS1		NAT4			
					PEG		A1	
Backbone	Prefiltering	Mode	CC_{raw}	CC_{norm}	CC_{raw}	CC_{norm}	CC_{raw}	CC_{norm}
2D-CNN	None	Single	47.2	65.1	35.2	48.5	41.7	56.8
		Population	**47.9**	**66.2**	**36.0**	**50.5**	**43.4**	**60.1**
	AdapTrans	Single	48.3	66.7	37.9	52.9	44.3	60.9
		Population	<u>**50.0**</u>	<u>**69.2**</u>	<u>**39.2**</u>	<u>**55.2**</u>	<u>**46.4**</u>	<u>**64.5**</u>

This very positive option is now discussed in a new section of the results ('*Further performance improvements with an augmented AdapTrans scheme and neural population training*').

p. 8. "Optimization process." Can the authors clarify if the AdapTrans-specific parameters (w, a) were fit simultaneously with the other model parameters? And separately for each neuron? The language, especially around Eq. 5 is a bit confusing. Does Eq 5 indicate the initial values of a (tau?) for all models fits?

AdapTrans-specific parameters (w, a) were first initialized with a biologically motivated logarithmic distribution (Eq. 5, Dean et al., Willmore et al.) and were then fitted simultaneously alongside the model backbone, with the same optimizer, learning rate, etc.

We have clarified this point at several places of the Materials and Methods, namely the ***A frequency-wise application*** subsubsection, the ***Integration within larger models of audition*** subsubsection, and the ***Optimization Process*** subsubsection.

p. 8. "Cross validation methodology" section. It appears that different test sets were used, but the number of trials varied across stimuli, and in some cases there was only one repeat. How was CCnorm computed for the single trial data?

That is correct. For single trial data, in order not to overestimate the performances of the models, we assumed the worst case scenario of *CCnorm = CCraw*, as indicated in the *"Materials and Methods"* section, *"Task and Evaluation of performance"* subsection, *"Correlation coefficient between recorded and predicted responses"* subsubsection (p. 9). This is equivalent to having neural recordings that are identical across trials, therefore with a Signal Power (SP) of 1 in Schoppe et al. 2016, or a CCmax of 1 (Hsu et al. 2004). As a

result, for these data, we provide a lower bound of the performances of the models. We have modified the associated paragraph in this new version to clarify this point.

Note that we have modified the way we estimate the CCnorm values, following suggestions from reviewer 2.

p. 8. Table 2. Please double check numbers. Eg, for the NAT4 dataset, the number of units does not match the numbers on p. 7. Also "3-63 x 1-25" for the Wehr dataset is difficult to parse.

We apologize for this mistake and for this lack of clarity. We have reworked the part of the "Materials and Methods" related to datasets and hope it is clearer now.

p. 10 "… offsets of different frequency components with a rich spectral stimulus" It is not clear what this means.

Thanks for pointing that out. We rephrased it for: *"Auditory neurons in areas MGB and AAF of the mouse auditory cortex can detect the offsets of different frequency components within complex structures of sound stimuli."* We hope that it clarifies.

p. 14. "… previous studies" The authors might also consider work by Lopez Espejo et al (PLoS CB 2019) that incorporated short-term synaptic plasticity into LN models, which also could account for sustained + transient responses (though did not distinguish between on and off). Additionally, work by Gill et al (JCN 2006) incorporated cochlear adaptation into LN models.

Thank you for indicating these extremely relevant studies. They are now cited and briefly discussed in the new version of the manuscript:

- in the discussion for both Lopez Espejo et al. and Gill et al.
- also in the *Introduction* and *A frequency-wise application* subsubsection within the *Materials and Methods* section for Gill et al.

New references added:

- Lopez Espejo, M., Schwartz, Z. P., & David, S. V. (2019). Spectral tuning of adaptation supports coding of sensory context in auditory cortex. *PLoS computational biology*, *15*(10), e1007430.
- Gill, P., Zhang, J., Woolley, S. M., Fremouw, T., & E. Theunissen, F. (2006). Sound representation methods for spectro-temporal receptive field estimation. *Journal of computational neuroscience*, *21*, 5-20.

Reviewer #2

Reviewer #2: This paper provides a model of neural responses in auditory cortex which builds on previous models, explicitly incorporating sustained and transient ON and OFF responses. The model provides better performance on some data sets than previous models. This is an interesting problem, and the authors have presented a nice new model which appears to be a useful step forward in pragmatic, biologically plausible computational modelling of neurons in mammalian auditory cortex. However, I have a number of concerns which make it hard to compare the performance of the new model with other similar models. I am also concerned that some claims are misleading or unjustified. Additional analyses are needed to address these concerns.

1. The authors introduce a new measure of prediction quality -- cc_norm that is normalised on a per-neuron and per-stimulus basis. This makes it hard to compare with previous work. The authors do not clearly explain why they did this. Using this measure makes it hard to understand whether the authors' models have been fitted correctly, and how these results compare with previous efforts. For each stimulus set they use, the authors should provide a direct comparison with the original authors' models, using the prediction quality metric used by the original authors. If the authors want to use a new metric, they should explain clearly what they believe are the advantages of the new metric, and provide quantitative justification for this.

Following the suggestion of this reviewer, we now use the method described in Schoppe et al. (2016) for calculating the CCnorm values. We still normalize on a per-neuron basis but now across all stimuli of the same set (i.e., train, validation, test). Importantly, this modification only has a weak impact on the estimated values and does not change our main results, which is that using our approach improves neural fitting performances.

2. The authors make comparisons between their model and a similar model which the authors refer to as Willmore et al (2016). However, this comparison is quite misleading because the "Willmore et al (2016)" model used here has several important differences from the model that was introduced in Willmore et al (2016). These differences are highly likely to affect the performance of this model, and so it is incorrect (and misleading) to present the authors' results as a test of Willmore et al's model. The authors say that they failed to replicate Willmore et al's results. This is likely explained by the differences between the model that they tested and the actual model used by Willmore et al. To fix this, the authors should implement Willmore et al's model accurately. The differences are listed below. Alternatively, they should make it clear that the model they are testing has some similarities with Willmore et al but is not the same model, and remove claims about failing to reproduce Willmore et al's results.

We agree with this reviewer that we did not exactly reproduce the work described in Willmore et al., (2016). One of the points of our study is to provide a general framework (i.e., all the data, pre-processing and processing codes under the same format that is compatible with Pytorch) to compare the performances of different models of the auditory cortex. In our

opinion, a fair comparison between two models can only be obtained if they are implemented using the same pipeline (i.e., using the same regularization methods, fitting approaches, number of cochleogram channels, ….). Otherwise, it is impossible to know whether a model with higher performances is genuinely better than another, or if it's rather its implementation pipeline that is more efficient.

In this new version, we made it clear that we did not reproduce the entire pipeline described by Willmore et al. (2016) but that we rather reimplemented their IC adaptation filter. Importantly, we have modified this reimplementation since our first submission and it now includes the half-wave rectification, which strongly increases the performances (see our response to point 4 below).

3. Similarly, the NRF and DNet models as implemented here are not identical to the models used by the respective authors. In particular, the use of Sohl-Dickstein's sum of functions optimiser was important for these models (https://github.com/Sohl-Dickstein/Sum-of-Functions-Optimizer). Again, this means that the authors should not claim that their results are representative of those models.

As reported in our response to the previous point, one of the objectives of our study is to provide a fair comparison between models. For that, we believe it makes more sense to use a general implementation pipeline. Our optimization is based on the Adam optimizer which is very commonly used in the machine learning community (see Kingma and Ba, 2014, a study that collected more than 175,000 citations), natively implemented on Pytorch and which was shown to provide accurate results.

In this new version, we have clarified that we did not reproduce the whole pipeline of the study describing the NRF and DNet models (Rahman et al., 2019) but rather that we reused the associated models. This is done in a new paragraph of the '*Task and Evaluation of Performance*' subsection.

New reference added:

- *Kingma, D. P., & Ba, J. (2014). Adam: A method for stochastic optimization. arXiv preprint arXiv:1412.6980.*

4. AdapTrans has key similarities with the model presented here as "Willmore et al (2016)" (hereafter MWEA for "modified Willmore et al"). Yet the two models perform very differently here, with AdapTrans doing very well, and the MWEA doing very poorly (being beaten by an LN model). Additional analysis is needed to uncover what is going on here. Why does the MWEA fail to improve on the LN model? What are the key properties of AdapTrans that make it so much better than the MWEA? The authors need to unpick exactly what differences between the two models (and the original Willmore et al model) are responsible for the differences in predictive power.

We thank this reviewer for pointing out that the performances obtained with our previous implementation of IC adaptation (i.e., the model described by Willmore et al., see our response to point 2 above) were poor. We realized that we had wrongly interpreted the equation provided in their figure 1 (see their panel B): we had implemented an absolute

value instead of a half-wave rectification. We apologize for this mistake, which was corrected in our new submission. IC adaptation now improves LN models (see the new version of table 4) on the NS1 dataset (on which it was originally developed by Willmore et al.) as well as on the Wehr dataset, but not on NAT4. Its performances are also more comparable to those obtained with AdapTrans on NS1. Importantly, this modification leaves our previous results unchanged as AdapTrans (almost) systematically leads to better performances than IC adaptation for all the models and datasets (the only exception being for the 2D-CNN model on NS1), in part because AdapTrans, but not IC Adaptation, captures OFF-responses (see Fig. 6C)

We have clarified these points in the new version of the discussion (we have notably modified the paragraph on the comparison between AdapTrans and IC adaptation) and hope that we now provide a fairer description of the performances of the two approaches.

5. The authors make the very surprising claim that regularisation is not required when fitting linear models to physiological data. This is at odds with previous literature, which the authors cite. I suspect that this is a mistake, and the authors methods are somehow introducing regularisation (e.g. by early stopping). This claim needs to be explained in much more detail, evaluated carefully to see if it is really correct, and, if so, it needs to be quantitatively justified.

Thanks for this comment. It is true that we used early stopping and that it constitutes a form of regularization. Additionally, our use of Batch Normalization [Ioffe and Szegedy (2015)] could be considered as yet another form of regularization. We have clarified this point in the new version of the manuscript and removed claims supporting the idea that regularization is not needed in our approach. Note that beyond early stopping and BN, we did not apply any other form of regularization classically used in the auditory STRF literature. We investigated whether weight decay (a L2 penalty readily implementable in our PyTorch setup) upon spectro-temporal weights could improve performances. In particular, we trained our models on NS1, tried two different values of this hyperparameter (wd=0.01 and wd=0.1), and found the test CCs [%] reported in the table below, for comparison with those reported in our submission.

**Model**		**NS1 test set**			
		varied (ours)		fixed (Rahman et al.)	
Backbone	Prefiltering	CCraw	CCnorm	CCraw	CCnorm
L	Control	35.9	49.3	**40.1**	**52.0**
	AdapTrans	43.1	59.2	**47.0**	**61.0**
LN	Control	**33.7**	**46.6**	32.6	42.2
	AdapTrans	43.3	59.6	**46.6**	**60.3**
NRF	Control	43.7	**60.4**	**45.8**	59.5
	AdapTrans	46.6	64.3	**49.6**	**64.4**
DNet	Control	34.0	46.7	**39.3**	**50.1**
	AdapTrans	44.4	61.3	**47.9**	**62.2**
2D-CNN	Control	47.2	65.1	**47.5**	61.4
	AdapTrans	48.3	**66.1**	**50.8**	66.0

As can be observed, using a L2 penalty on the weights of our models had little impact on performances. This result is in line with what was reported by Pennington and David (2023,

see their ‘Model optimization’ section on pages 19-20: “*We also tested dropout and L2 regularization, but we found little to no benefit and chose to exclude them from this study for simplicity.*”).

To clarify this point, we added a new paragraph on regularization in the discussion. We also provide the table above in the supplements (see supplementary section *“Influence of weight decay on model performance”*).

Major concerns

Pg 6. The authors make surprising claims that regularisation is not needed for fitting linear models. The previous literature, which they cite, has usually found that regularisation is critical when fitting these models to physiological data sets. This deviation from previous work is controversial. The authors say that regularisation "did not necessarily prove beneficial in terms of performance in our setup". This is insufficient justification for a controversial claim. I think the authors need to re-investigate this, and establish whether it is really true that no regularisation is being used here. One possibility is that the authors are effectively using early stopping (which can be seen as a form of regularisation) by using too few epochs to train their models. The authors speculate that their use of gradient descent might make regularisation unnecessary. There is some literature on this idea (e.g. https://arxiv.org/abs/2009.11162). I think detailed quantitative analysis is needed here, to explain how the models are avoiding overfitting.

See our response to the previous point (i.e. point 5).

Pg 8. The authors normalise cc_norm on a per-stimulus basis as well as per-neuron. The authors justify this as follows: "we decided to normalize per neuron and per stimulus, as there can be a variability in the responses depending on both of these factors". It is true that cc_norm is going to depend on both neuron and stimulus; however, it is not clear why or if the stimulus-based variability should be excluded. In the limit of short stimuli, normalising on a per-stimulus basis will lead to excluding all of the stimulus-based variability, and this approach will therefore tend to exaggerate cc_norm values. I think the authors need to explain why they think this approach is superior. They also need to provide analysis to quantitatively justify this non-standard choice, by showing why this is better than the standard approach of normalising only on a per-neuron basis (or per-neuron and per stimulus class). An alternative would be to use previously published measures of prediction performance.

We understand this reviewer concern about the metric used to evaluate neural fitting performances. In order to facilitate the comparison with previous studies and following his/her suggestion (see one of the next points below), we now use the method described in Schoppe et al. (2016), which is more standard and more accurate, for calculating the CCnorm values. In particular, we no longer normalize on a per-stimulus basis. The

associated results are shown in the new version of Table 4. Note that this modification only has a minor impact on the obtained values. Importantly, it leaves unchanged the main result of our study which is that using AdapTrans on a specific model enhances its neural response fitting performances. These improvements in CCnorm with AdapTrans are in line with those observed with the other metrics (i.e., CCraw and the coherence values that we added in this new version following a suggestion from reviewer 1).

New reference added:

- Harper, N. S., Schoppe, O., Willmore, B. D., Cui, Z., Schnupp, J. W., & King, A. J. (2016). Network receptive field modeling reveals extensive integration and multi-feature selectivity in auditory cortical neurons. *PLoS computational biology*, *12*(11), e1005113.

Pg 7. The use of an unusual measure of prediction performance makes it difficult to compare model performance with previous efforts. In order for the reader to be able to understand that the authors' models have been fitted correctly, and how they compare with previous efforts, for each previous data set used, the authors should provide direct comparisons with previous models used by the authors of that dataset, using the same measures of prediction performance as the original authors. It is fine to use the new measure as well, if it is justified as described above.

As reported in our response to the previous point, we now use the method described by Schoppe et al. (2016) to define the CCnorm values.

Pg 8. Calculating cc_norm via cc_half is no longer the best approach. It makes sense to use this measure for comparison with Willmore et al (2016), which used the same method. However, I would recommend using the methods in Schoppe et al (2016) for calculating cc_norm in general.

See our responses to the two previous points. Note that we have preferred to compute the CCnorm values with the method described in Schoppe et al., (2016) rather than that in Willmore et al. (2016) because this approach is generally better. In our opinion, it does not prevent from comparing the performances obtained with IC adaptation (the model of adaptation described by Willmore et al.) and AdapTrans as the same metric is used in both approaches (see our response to the second point of this reviewer). We have also included this metric on our online code repository.

Pg 9. The authors claim that Rahman et al (2019) overestimated the cc_norm of models. Rahman et al used an approach where a dataset was held out <u>during the whole process of model development,</u> <u>and only 'opened' once the final models had been selected and trained</u>. This approach has a particular benefit: the model hyperparameters are not overfitted to the final test set. This means that this approach is likely than other approaches, a priori, to more accurately estimate CC_norm, because the model hyperparameters have not been accidentally overfitted (through an iterated process of model development and testing) to the test set.

I would suggest that the authors either provide figures and numbers concretely demonstrating their case that the overestimation occurs, and more clearly state the benefit of the Rahman et al. approach, as well as its cons. Alternatively, I would drop this paragraph because it makes an argument that is not supported by data.

We acknowledge that we had actually misunderstood the term '*held out*' in Rahman et al. study, and we thank the reviewer for this clarification. Only using the test set at the very end of the model development and data analysis process greatly alleviates our concerns for overfitting and we have therefore updated this paragraph as required.

We nonetheless believe that keeping a unique test set, even held-out, can be dangerous when the dataset is small, such as in the present case (14 clips for training, 2 for validation, 4 for test). Indeed, the chosen test could be much easier than the average, and since only the performances of the former are evaluated, it can potentially lead to overestimated results. This is something we noticed with the test set used by Rahman et al. In order to quantitatively support this claim, we set Rahman et al's test set apart (4/20 stimuli) and retrained the same models according to our method, averaging the test performances obtained with 10 different train/validation splits of the remaining data. Since we did not use Rahman et al's test set during model development, we can indeed consider it "held out" here. The table below reports the performances (CCnorm) obtained in these conditions.

**Model**		**NS1 test set**			
		varied (ours)		fixed (Rahman et al.)	
Backbone	Prefiltering	CCraw	CCnorm	CCraw	CCnorm
L	Control	35.9	49.3	**40.1**	**52.0**
	AdapTrans	43.1	59.2	**47.0**	**61.0**
LN	Control	**33.7**	**46.6**	32.6	42.2
	AdapTrans	43.3	59.6	**46.6**	**60.3**
NRF	Control	43.7	**60.4**	**45.8**	59.5
	AdapTrans	46.6	64.3	**49.6**	**64.4**
DNet	Control	34.0	46.7	**39.3**	**50.1**
	AdapTrans	44.4	61.3	**47.9**	**62.2**
2D-CNN	Control	47.2	65.1	**47.5**	61.4
	AdapTrans	48.3	**66.1**	**50.8**	66.0

We observe that in terms of CCraw, performances are almost systematically better on Rahman's test set. In terms of CCnorm, the Rahman et al. 's method also yields better values on average. These increases can be quite significant, especially for the L model.

Please note that it is possible for a given model to have lower CCnorm than another, despite having better CCraw, depending on the intrinsic variability of the responses that the first model better fits. E.g., if model A has much better CCraw than model B on very reliable responses, the CCnorm of model A will not increase much. In addition, the metrics reported with our method are the average of 10 random seeds, so there are many possibilities where this could happen.

In conclusion, we showed here that keeping a fixed test set can be a dangerous evaluation strategy and results in over- or underestimated performances. We have clarified this point in

the new version of the manuscript; the table above is now provided in the supplements (see Supplementary Note 4).

Pg 9. The authors say that previous public modelling tools such as NEMS are outdated. This is untrue, and this claim should be removed. The NEMS toolkit is under active development (last github commit was in February 2024).

We thank this reviewer for this information. We have modified our description of NEMs library in the new version of the manuscript.

Pg 9. "Indeed, in absolute and with a well normalized CC, a CCnorm of 1 is reachable; it is only a matter of data-preprocessing and model –the latter guided by knowledge in neuroscience and machine learning– to bridge the remaining gap." I think this claim should be removed, because it is speculative. Achieving a cc_norm of 1 would require models that are substantially better than any that exist today. It would also be necessary to be able to fit those models perfectly using finite, limited, noisy physiological data sets. It is not at all clear that this is achievable.

We thank the reviewer for this comment. We removed this claim in the new version of the manuscript.

Pg 12. The modified Willmore et al model (MWEA) does very poorly in this comparison, despite having important similarities with AdapTrans. Specifically, it appears to perform worse than an LN model, despite Willmore et al finding that it was useful. This raises two key issues:

1. There are important differences between the MWEA model and the original Willmore et al model, which likely explain the poor performance of the MWEA here. The ones I can think of are:

**** Missing half wave rectification***

**** No regularisation***

**** Different fitting method (gradient descent vs GLMnet)***

**** Adaptation time constants are learned***

**** Different performance metric (ccnorm normalised per-stimulus)***

Also, these may differ, depending on stimulus set:

**** Cochleagram algorithm (gammatones vs spectrogram)***

**** Different number of cochleagram channels***

As reported in our response to the point 4 of this reviewer (see above), we have identified what was wrong in our previous implementation of IC adaptation. We have

corrected the problem and this approach now systematically improves LN models (see the new version of Table 3) on NS1 and Wehr, but not NAT4 datasets, on which it performs worse than the raw spectrogram itself. Even with this correction, we agree that there are still differences between our implementation and the processing pipeline described by Willmore et al. but, as we wrote it in our response to point 2 of this reviewer (see above), we think that the better way to compare two approaches is to implement them using the same pipeline, which is what we do here.

As reported above, we have clarified these points in the new version of the manuscript and hope that it provides a better view of the performances obtained with IC adaptation and AdapTrans.

The results of the MWEA should not be presented as representative of the Willmore et al model. Also, the authors should do a hierarchical model comparison, adding features to the model one by one, to identify which features of the two models are important for prediction quality.

As mentioned above, in the new version of the manuscript, we have clarified that our study uses a reimplementation of IC adaptation and that our pipeline is not identical to the one described by Willmore et al. Since our corrected version of this reimplementation (see above) now provides results that are in line with what was reported in the original study (notably the fact that IC adaptation systematically improves the LN model on NS1 dataset), we think that the hierarchical model comparison suggested by this reviewer is not needed anymore.

2. The MWEA and AdapTrans are quite similar to each other. It is surprising that the MWEA does so badly, and AdapTrans does so well. Again, the authors should do a hierarchical comparison to understand what features of the two models are important for the differences in prediction performance.

See our responses above.

The authors should provide full equations for their model.

We are concerned about the reviewer's comment, but are not sure about which type of equation he/she is referring to. The full equations for AdapTrans are given in the *"AdapTrans model of auditory ON-OFF responses and adaptation"* subsection of the *"Materials and Methods"*, and additional properties such as its recurrent formulation are provided in the *"Supplementary Note 1: Mathematical derivation of AdapTrans properties"*.

Because all the models of the auditory cortex that we use in our study (L, LN, NRF, DNet and 2D-CNN) were fully characterized in previous publications, we only remind their main properties in the *Materials and Methods"* section and do not provide their complete equation to avoid an unnecessary increase of the manuscript length. We have clarified this point in the new version.

Minor concerns

Title and elsewhere: The paper claims to present a general theoretical framework, but the contribution is about incorporating known aspects of biology into computational models, to increase the predictive power of the models. This is a worthwhile endeavour, but not a theoretical one. I would rewrite the title to accurately reflect this, and remove claims about a theoretical framework in the main text.

AdapTrans is a *high-level, descriptive* model of ON-OFF responses and adaptation in the auditory brain, as opposed to a *mechanistic* one (cf. Discussion), which is perhaps indeed what is considered as "*theoretical"* in most cases. We would like to remind that our study not only shows that AdapTrans makes general response models more accurate, but also that it can account for auditory responses *on its own*. That being said, to avoid any such confusion as to the meaning of the word "*theoretical"*, we have replaced "theoretical" by "model" in the title and adapted our previous claims, as demanded by this reviewer.

Pg 9. The authors emphasise their provision of code and data for their model, as a model of scientific reproducibility. I agree that it is good that these are provided. However, this is not unusual in 2024, and is required by many funding bodies and by PLoS Comp Biol. This therefore does not deserve the emphasis given in the paper. The commentary about this should be removed, and the authors should simply provide links to the required repositories.

This reviewer is right to remind that fortunately, providing codes and data for a model is now the norm. In this regard, we agree that providing the code for AdapTrans is the least we can do. However, contrary to a majority of studies in this field, we not only provide the code for *our own* model, but also re-implementations of *others*, for a fair comparison across them in a single, unified framework based on an unique pipeline. Besides AdapTrans, one major contribution of our study is this systematic evaluation across datasets and models, as well as the associated library, which we hope will foster new standards and accelerate the evaluation of computational methods. We have clarified this point in the new version of the manuscript.

There are a number of typos -- e.g. "Mcdermott" should have a capital D, "errobars" should read "error bars", "spectogram" should read "spectrogram", "euqla" should read "equal, "black-box" should read "black boxes", "adress" should read "address". The whole MS should be spellchecked.

We apologize for these typographical errors and thank this reviewer for pointing them out. We have corrected them in the current version of the manuscript.
